# Does computed tomography angiography for the Adamkiewicz artery add value over standard scanning in thoracic endovascular aortic repair planning?

**DOI:** 10.1016/j.xjse.2025.100043

**Published:** 2025-01-13

**Authors:** Tatsuya Nishii, Hiroki Horinouchi, Akiyuki Kotoku, Yojiro Koda, Rina Sakai, Yuna Okura, Tomoro Morikawa, Yasutoshi Ohta, Hitoshi Matsuda, Tetsuya Fukuda

**Affiliations:** a Department of Radiology, National Cerebral and Cardiovascular Center, Suita, Osaka, Japan; b Department of Vascular Surgery, National Cerebral and Cardiovascular Center, Suita, Osaka, Japan

**Keywords:** computed tomography angiography, endovascular aneurysm repair, spinal cord ischemia, aortic aneurysm—thoracic, aortic dissection

## Abstract

**Objectives:**

To determine whether Adamkiewicz artery (AKA)-specific computed tomography angiography (CTA) offers additional value for thoracic endovascular aortic repair (TEVAR) planning beyond standard CTA and to identify cases that might benefit from its use, particularly in influencing treatment decisions and assessing spinal cord ischemia (SCI) risk.

**Methods:**

We retrospectively reviewed 176 consecutive patients who underwent both standard CTA and AKA-CTA. Patients with >90 days between CTAs and those with reduced-contrast protocols were excluded. Two radiologists assessed the AKA branching levels, and the inter-CTA agreement of AKA findings on standard CTA was measured using AKA-CTA as a reference. We evaluated changes in TEVAR planning and SCI risk scores on the basis of the additional AKA-CTA data and conducted a multivariate logistic analysis to identify predictors of these changes.

**Results:**

Among 160 cases (median age 70 years [interquartile range, 58-77]; 110 male patients), the AKA was identified in 95 (59.4%) by standard CTA and in 155 (96.9%) by AKA-CTA. Standard CTA demonstrated 50% concordance with AKA-CTA findings. TEVAR planning was performed in 57 of 80 cases with inconsistent AKA results between CTAs, with adjustments in 18 cases on the basis of AKA-CTA information. Distal landing zone of ≥5 was the only significant predictor of clinical decisions influenced by AKA-CTA (odds ratio, 16; 95% confidence interval, 3.8-111).

**Conclusions:**

Although standard CTA identified the AKA with 50% concordance to AKA-CTA findings, AKA-CTA provided valuable insights over standard CTA, particularly in cases with a distal landing zone of ≥5, significantly impacting TEVAR planning and SCI risk assessment.

## Graphical abstract

**Figure:**
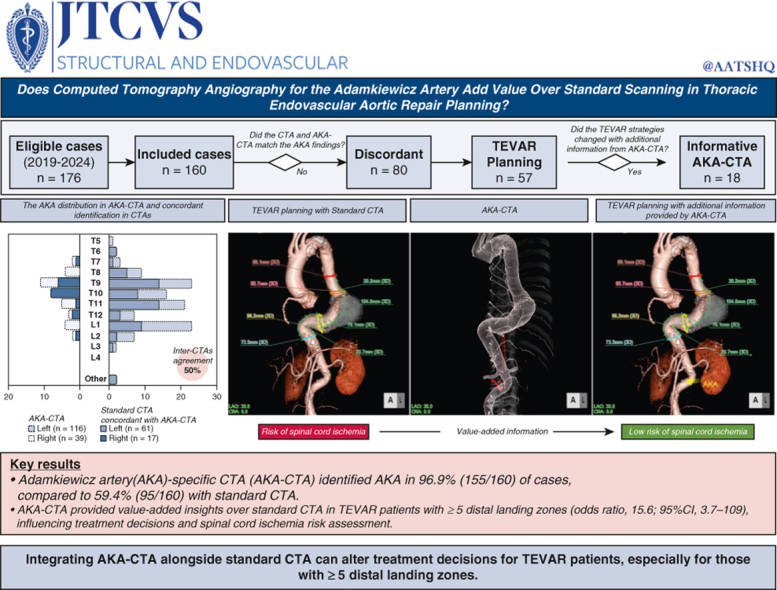

**Figure:**
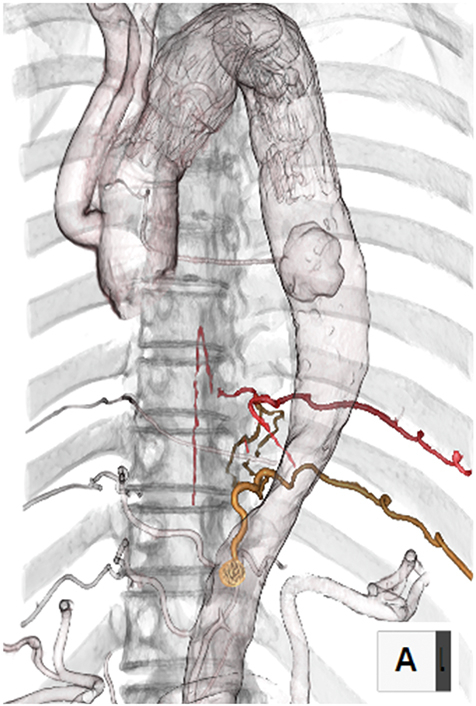
Adamkiewicz artery−specific CTA provided value-added insights over standard CTA.

**Central Message**

Adamkiewicz artery−specific CTA provided valuable information beyond standard CTA in thoracic endovascular aortic repair patients with ≥5 distal landing zones, thereby influencing decision-making.

**Perspective**

The Adamkiewicz artery (AKA)-specific CTA can effectively identify the AKA; however, its influence on decision-making for thoracic endovascular aortic repair (TEVAR) strategy is not well understood. Our study revealed that incorporating AKA-CTA with standard CTA can alter treatment decisions for patients who undergo TEVAR, particularly for those with ≥5 distal landing zones.

Reconstruction of the segmental artery supplying the Adamkiewicz artery (AKA) is essential in mitigating the risk of spinal cord ischemia (SCI) during surgical aortic repair.^1^, ^2^, ^3^ Thus, identifying the AKA is crucial before descending aortic open surgery.^2^ However, preoperative identification of the AKA in thoracic endovascular aortic repair (TEVAR) remains controversial,^4^, ^5^, ^6^, ^7^, ^8^ and current guidelines do not mandate its identification.^9^^,^^10^ In a study involving >200 patients,^7^ the authors reported a greater incidence of SCI when the AKA was covered during TEVAR, although this difference was not statistically significant. Known risk factors, such as procedure duration and iliac access, were substantial predictors of SCI only when the AKA was compromised. These findings suggest that covering the AKA may indirectly reduce spinal cord blood flow during TEVAR and that preserving the AKA may be preferable for at-risk patients.^7^

Accurate identification of the AKA by computed tomography angiography (CTA) requires high spatial resolution and sufficient contrast as the result of its anatomical features.^11^^,^^12^ Advances in computed tomography (CT) technology over the past few decades have increased the identification rate of AKA by using AKA-specific computed tomography angiography (AKA-CTA) to 90% to 96%.^13^, ^14^, ^15^ AKA-CTA is now a justified, noninvasive alternative to spinal angiography for assessing the spinal blood supply before aortic repair in addition to the standard CTA. Meanwhile, the AKA is occasionally visible on the standard CTA. In addition, a high-resolution CT system can detect AKA in approximately one half of the cases, even with standard CTA.^16^ As the result of CT system limitations, generalizing the results is premature. Thus, whether standard CTA can replace AKA-CTA, or how much additional information AKA-CTA provides over standard CTA, remains unclear. This extra examination could be added if the AKA-CTA findings significantly influenced the planning or SCI risk assessment during TEVAR.

Therefore, we hypothesized that selecting AKA-CTA over standard CTA would maximize its value in optimal patients. This study aimed to determine whether the value-added information provided by AKA-CTA beyond standard CTA influences TEVAR strategies and to identify the patients who would benefit most from this additional procedure.

## Methods

### Study Population

We retrospectively analyzed data from 176 consecutive patients who underwent standard CTA and AKA-CTA before aortic repair between January 2021 and February 2024. When considering therapeutic intervention in the descending aorta, we routinely assessed the AKA using AKA-CTA and standard CTA. Furthermore, our institution performs >150 endovascular aortic repairs annually with experienced multidisciplinary teams, classifying it as a high-volume aortic center.^9^ We excluded patients with a CTA-to-AKA-CTA interval of >90 days and those using reduced contrast protocols as the result of renal impairment. The institutional review board of the National Cerebral and Cardiovascular Center approved this retrospective study (approval number: R19039-3, date: March 8, 2022) and waived the requirement for written informed consent because of its retrospective nature.

### Image Acquisition and Reconstruction

Standard-body CTA was performed using 3 CT scanners: 320-row Aquilion ONE (Canon Medical Systems), 256-row Revolution CT (GE HealthCare), and 196-row SOMATOM Force (Siemens). A contrast dose of 18.5 mgI/kg/s was used, with image acquisition on the basis of bolus tracking. Images were reconstructed using deep learning (AiCE, Canon; TrueFidelity, GE) or iterative methods (ADMIRE; Siemens) with 1- to 1.25-mm slice thickness and 400-cm field of view. We used the 196-row SOMATOM Force (Siemens) in dual-power mode with low-kV potential for AKA-CTA. Noncontrast, early, and late arterial phase scans covered the aortic arch to L4. Contrast was administered at 26 mg I/kg/s, optimized for peak contrast at the T10 level. Images were reconstructed with a 160-cm field of view, 0.75-/0.5-mm thickness/interval, and a sharp filter kernel. Bone subtraction images were created using Ziostation2 (Ziosoft). The detailed imaging methods are described in Appendix E1 and Tables E1 and E2.

### Image Analysis

Two board-certified radiologists (T.N. and A.K., with 15 and 10 years of experience, respectively) assessed AKAs in the arterial phase of standard CTA and the early and late arterial phase images, including bone-subtracted AKA-CTA images by consensus. Identification relied on a published stepwise approach and a 6-point scoring system that assessed hairpin vessels, their continuity with the aorta, and vessel attenuation washout during the late arterial phase.^14^^,^^15^ A total score ≥3 indicated positive AKA identification. We evaluated the pathway from the aorta to the AKA as the connecting segmental artery by noting its origin and location. Only hairpin vessels and continuity scores were obtained by standard CTA because a late arterial phase was absent, with ≥2 as the threshold for positive AKA identification. We evaluated standard CTA and AKA-CTA images at least 1 month apart. Two radiographers (Y.O. and R.S., with 1 and 2 years of experience) and a radiologist (T.N.) assessed the quality of arterial-phase images. The mean CT number and standard deviation in the aorta at the T5, 8, 12, and L3 were determined in circular regions of interest,^17^ ensuring avoidance of calcified areas and, in dissection patients, confirming measurements in the true lumen. The aorta's image noise and signal-to-noise ratio were determined as the means and standard deviation of the regions of interest, and the mean CT number was divided by the image noise.

The additional value of AKA-CTA was assessed in patients with discordant AKA results between CTAs. TEVAR planning was conducted through consensus reading by an interventional radiologist (H.H., with 12 years of experience) and vascular surgeon (Y.K., with 11 years of experience). We initially confirmed the suitability of TEVAR on the basis of information from the standard CTA and planned the optimal TEVAR for each patient. We selected GORE TAG conformable thoracic stent grafts (W.L. Gore & Associates). When information on the AKA was available from the standard CTA, the radiologist (T.N.) marked the segmental artery branches on the CT images. We determined the planned proximal and distal landing zones (LZs) and covering length. LZs were defined according to specific guidelines,^9^^,^^10^ with a focus on zones 4 and 5, which are bordered by the midpoint of the descending aorta. The distance between the left subclavian artery and the celiac artery was measured to determine the midpoint of the descending aorta. We simultaneously assessed the risk of SCI associated with the TEVAR plans using CTA. Each factor was scored as 1: coverage >200 mm,^4^^,^^18^ a history of aortic surgery,^19^ a "shaggy aorta,"^7^ obstructed spinal collateral source (left subclavian artery or hypogastric arteries),^8^ and the potential for AKA coverage.^4^, ^5^, ^6^, ^7^ The "shaggy aorta" was defined as an irregular atheroma with a luminal protrusion >5 mm thick covering more than one half the circumference of the descending aorta. When an AKA was not identified, TEVAR plans with a distal LZ ≥ 5 were considered to confer the risk of AKA coverage. We then examined whether the plan should be modified or whether the SCI risk score would change by adding AKA-CTA findings. The radiologist (T.N.) marked the AKA branching point on the TEVAR image and measured the distance from the celiac artery bifurcation. A plan or SCI risk altered because of additional data was regarded as AKA-CTA informative.

### Statistical Analysis

Continuous variables are expressed as median with interquartile range. All statistical analyses were performed using statistical software (R version 4.4; R Foundation for Statistical Computing).

We used Fisher exact tests to compare AKA detectability on standard CTA and AKA-CTA images. Subsequent analyses were performed on groups with concordant and discordant AKA diagnoses between CTA and AKA-CTA. These analyses included patient background, imaging parameters, objective image indices, and AKA findings using Mann-Whitney *U* and Fisher exact tests. The positive predictive value (PPV), negative predictive value (NPV), and concordance ratio of standard CTA for diagnosing AKA using AKA-CTA as a reference were also evaluated. The Wilcoxon signed-rank test was used to compare the radiation metrics and contrast doses between CTAs.

In cases with a TEVAR plan, we assessed differences in patient backgrounds, TEVAR plans, and SCI risk scores and whether AKA-CTA was informative, using Mann-Whitney *U* and Fisher exact tests. Multivariate logistic regression analysis identified the independent factors in patients with informative AKA-CTA findings. Finally, we compared the groups on the basis of the presence or absence of a sacrificed AKA with TEVAR.

## Results

**Figure 1:**
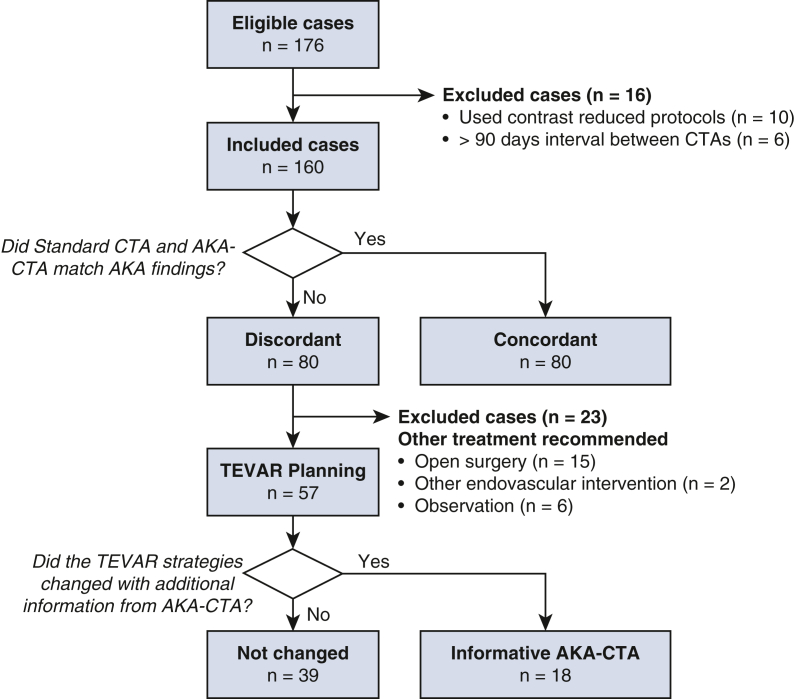
Flow diagram of the study. *CTA*, Computed tomography angiography; *AKA-CTA*, AKA-specific computed tomography angiography; *AKA*, Adamkiewicz artery; *TEVAR*, thoracic endovascular aortic repair.

**Figure 2:**
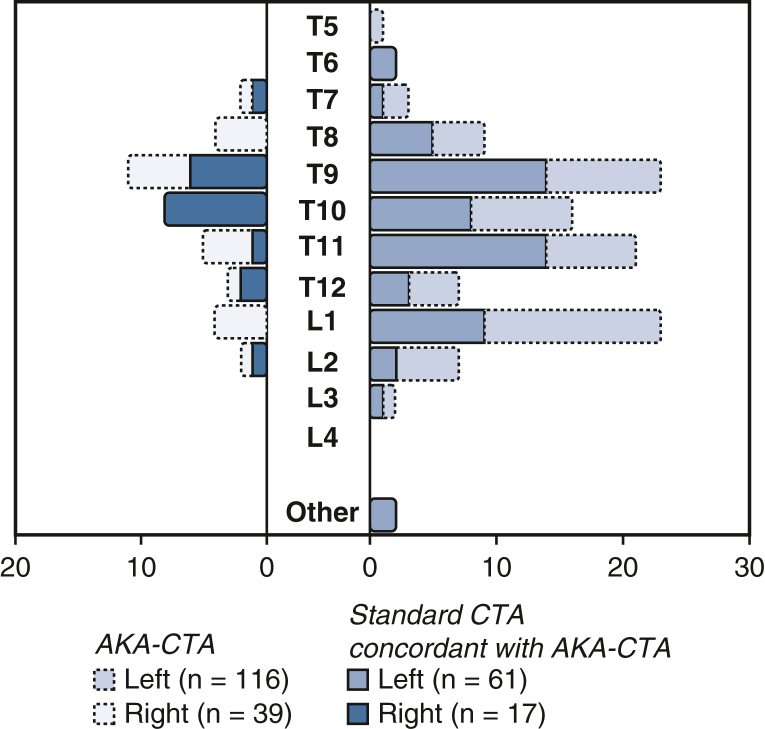
The Adamkiewicz artery distribution in AKA-CTA and concordant identification with standard CTA. *AKA-CTA*, Adamkiewicz artery-specific computed tomography angiography; *CTA*, computed tomography angiography.

Of the 160 patients (median age 70 [58-77] years, 110 male patients, all of Asian ethnicity), the aortic diseases included 105 dissections, 44 aneurysms, 4 intramural hematomas, and 7 others (Figure 1, Table 1). AKA-CTA identified AKA in 96.9% (155/160) cases. The AKA originated from the left segmental artery in 72.5% (116/160) of the cases and at the level of the eighth intercostal to the first lumbar artery in 84.8% (134/160) (Figure 2, Table E3).

**Table 1:** Patient demographics

Variables	Overall n = 160	Concordant n = 80	Discordant n = 80	*P* value
Age, y	70 (58, 77)	70 (58, 76)	71 (60, 77)	.54
Male patient	110 (68.8)	59 (74)	51 (64)	.23
Body mass index, kg/m^2^	23.2 (20.9, 25.7)	23.1 (20.5, 24.6)	23.4 (21.1, 27.1)	.12
Disease				.26
AD	105 (65.6)	56 (70)	49 (62)	
TAA	44 (27.5)	17 (21)	27 (34)	
IMH	4 (2.5)	2 (3)	2 (3)	
Other	7 (4.4)	5 (6)	2 (3)	
Interval between CTAs, d	12 (7, 20)	10 (6, 15)	13 (7, 22)	.14
Standard CTA parameters				
CT system				.64
CT1	30 (18.8)	15 (19)	15 (19)	
CT2	41 (25.6)	18 (23)	23 (29)	
CT3	89 (55.6)	47 (59)	42 (53)	
Urgent CTA examination	80 (50.0)	41 (51)	39 (49)	.87
High pitch scan	156 (97.5)	78 (98)	78 (98)	1.0
Tube potential, kV				.68
90	13 (8.1)	8 (10)	5 (6)	
100	75 (46.9)	37 (46)	38 (48)	
120	72 (45.0)	35 (44)	37 (46)	
CTDI_vol_, mGy	7.3 (5.8, 8.9)	7.3 (5.8, 8.9)	7.2 (5.8, 9.0)	.73
DLP, mGy × cm	497 (415, 677)	500 (414, 667)	497 (422, 688)	.60
Contrast amount, mg I/kg	372 (349, 410)	372 (353, 410)	372 (343, 410)	.30
Iodine delivery rate, g I/s	1.1 (1.0, 1.3)	1.1 (1.0, 1.3)	1.1 (1.0, 1.3)	.52
AKA-CTA parameters				
Tube potential, kV				.35
70	90 (56.2)	48 (60)	42 (53)	
80	41 (25.6)	21 (26)	20 (25)	
90	24 (15.0)	8 (10)	16 (20)	
100	5 (3.1)	3 (4)	2 (3)	
CTDI_vol_, mGy	16.3 (13.9, 23.0)	16.4 (13.9, 22.8)	16.3 (14.0, 23.2)	.75
DLP, mGy × cm	711 (607, 1022)	700 (611, 1000)	728 (601, 1063)	.62
Contrast amount, mg I/kg	508 (462, 539)	510 (474, 535)	506 (456, 539)	.46
Iodine delivery rate, g I/s	1.6 (1.4, 1.7)	1.6 (1.4, 1.7)	1.6 (1.4, 1.7)	.65

Data are shown as medians with interquartile ranges and numbers of patients n (%). *AD*, Aortic dissection; *TAA*, thoracic aortic aneurysm; *IMH*, intramural hematoma; *CTA*, computed tomography angiography; *CT*, computed tomography; *CTDI*_*vol*_, volume CT dose index; *DLP*, dose length product; *AKA-CTA*, AKA-specific CT angiography; *AKA*, Adamkiewicz artery.

**Figure 3:**
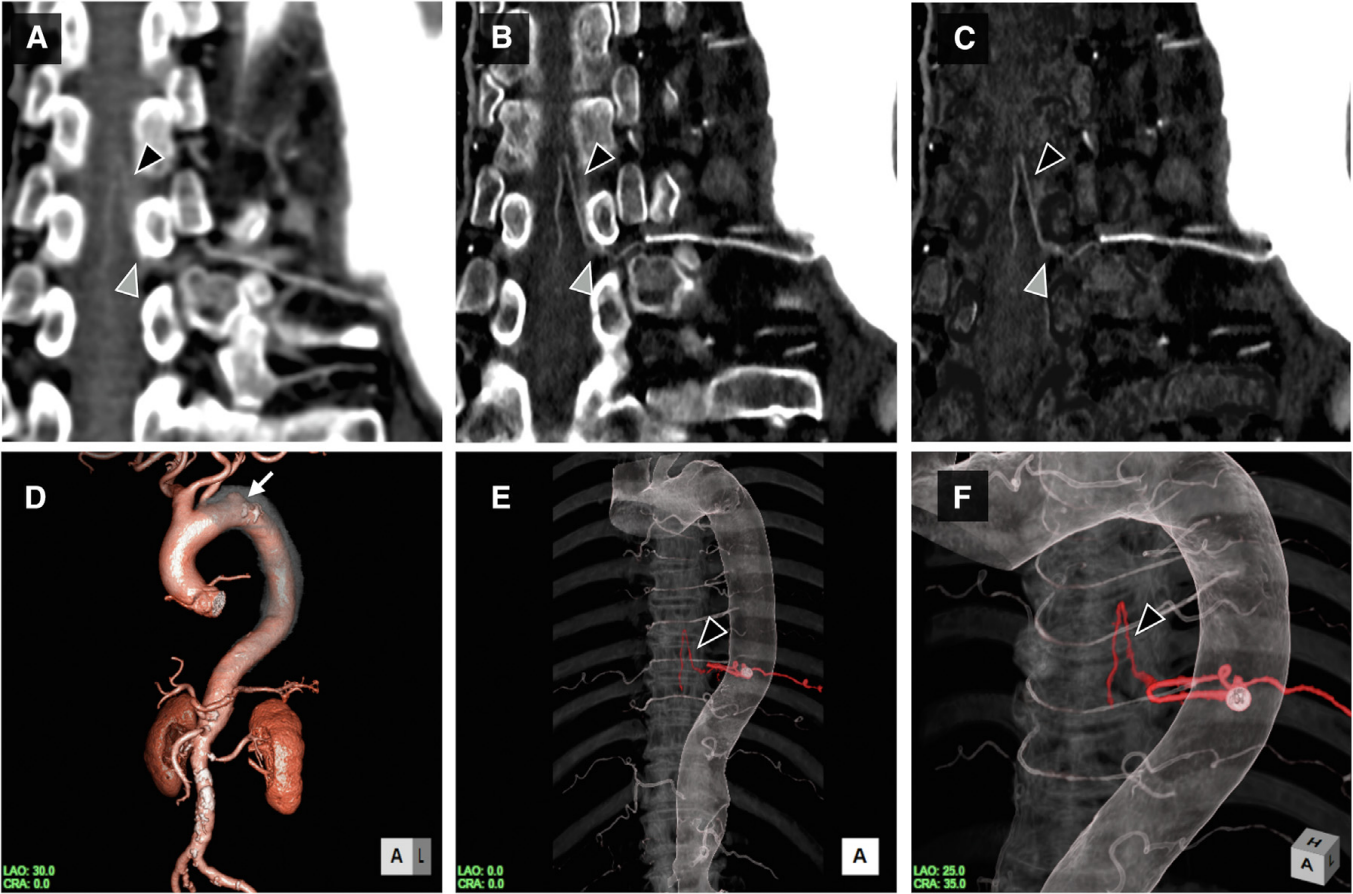
Representative case of concordant Adamkiewicz artery (AKA) identification in standard computed tomography angiography (CTA) and AKA-specific computed tomography (AKA-CTA) of an 85-year-old woman with aortic dissection. Curved multiplanar reformatted images of CTA (A), AKA-CTA (B), and bone-subtracted AKA-CTA (C), volume-rendered images of CTA (D), and AKA-CTA (E-F) are shown. The hairpin-shaped vasculature (*black arrowhead*) is the AKA, arising from the left T9th segmental artery. The continuity of the AKA in the intercostal foramen in standard CTA is equivocal (*gray arrowhead*) (A) but definitive in AKA-CTA (B, C). An ulcer-like projection at the proximal descending artery is indicated by a *white arrow* (D).

Standard CTA identified AKA in 59.4% (95/160) of the cases, which was lower than that of AKA-CTA (*P* < .001). Concordance between the CTA and AKA-CTA diagnoses was 50.0% (80/160). A typical case of both CTAs with concordant AKA identification is shown in Figure 3. Significant differences were noted between cases in which AKA originated from the lumbar level (Table 2). No notable differences were found in patient background and CTA imaging parameters. For AKA identification, the standard CTA had PPV of 82% (78/95) and NPV of 3% (2/65) compared with the reference AKA-CTA. The patients were exposed to significantly more radiation and contrast doses during AKA-CTA than standard CTA (*P* < .001 for all).

**Table 2:** Differences in imaging metrics and findings between AKA detection by standard CTA and AKA-CTA

Variables	Overall n = 160	Concordant n = 80	Discordant n = 80	*P* value
Quantitative image quality metrics				
CTA				
CT numbers of the aorta, HU	374 (327, 421)	381 (331, 417)	368 (318, 424)	.50
Image noise, HU	13.7 (11.0, 16.4)	13.6 (11.4, 16.5)	13.8 (10.7, 16.4)	.81
Signal-to-noise ratio	28.3 (22.8, 33.6)	28.3 (23.3, 33.2)	28.0 (22.0, 36.1)	.83
AKA-CTA				
CT numbers of the aorta, HU	759 (636, 898)	769 (661, 883)	751 (616, 930)	.67
Image noise, HU	33.3 (31.9, 35.1)	33.2 (31.9, 35.0)	33.5 (32.0, 36.1)	.30
Signal-to-noise ratio	21.9 (17.9, 27.7)	22.9 (18.9, 27.5)	21.0 (17.5, 27.8)	.30
AKA findings				
CTA				
AKA detection	95 (59.3)	78 (98)	17 (21)	<.001
Hairpin curve score, 1	121 (75.6)	79 (99)	42 (53)	<.001
Continuity score				<.001
0	66 (41.2)	3 (4)	63 (79)	
1	46 (28.7)	35 (44)	11 (14)	
2	38 (23.8)	33 (41)	5 (6)	
3	10 (6.2)	9 (11)	1 (1)	
Collateral pathway detection	10 (6.2)	10 (13)	0 (0)	.003
Other ARMA detection	8 (5)	6 (8)	2 (3)	.27
AKA-CTA				
AKA detection	155 (96.9)	78 (98)	77 (96)	1.0
Hairpin curve score, 1	159 (99.4)	79 (99)	80 (100)	1.0
Continuity score				.14
0	3 (1.9)	1 (1)	2 (3)	
1	10 (6.2)	4 (5)	6 (8)	
2	43 (26.9)	16 (20)	27 (34)	
3	104 (65.0)	59 (74)	45 (56)	
Washout score, 1	141 (88.1)	75 (94)	66 (83)	.05
Branching level, lumber	38 (23.8)	13 (16)	25 (31)	.04
Branching lumen, true	127 (79.4)	65 (81)	62 (78)	.44
Collateral pathway detection	24 (15.0)	10 (13)	14 (18)	.48
Other ARMA detection	23 (14.4)	6 (8)	17 (22)	.02

Data are shown as medians with interquartile ranges and numbers of patients n (%). *AKA*, Adamkiewicz artery; *CTA*, computed tomography angiography; *AKA-CTA*, Adamkiewicz artery−specific computed tomography angiography; *ARMA*, anterior radiculomedullary artery.

**Figure 4:**
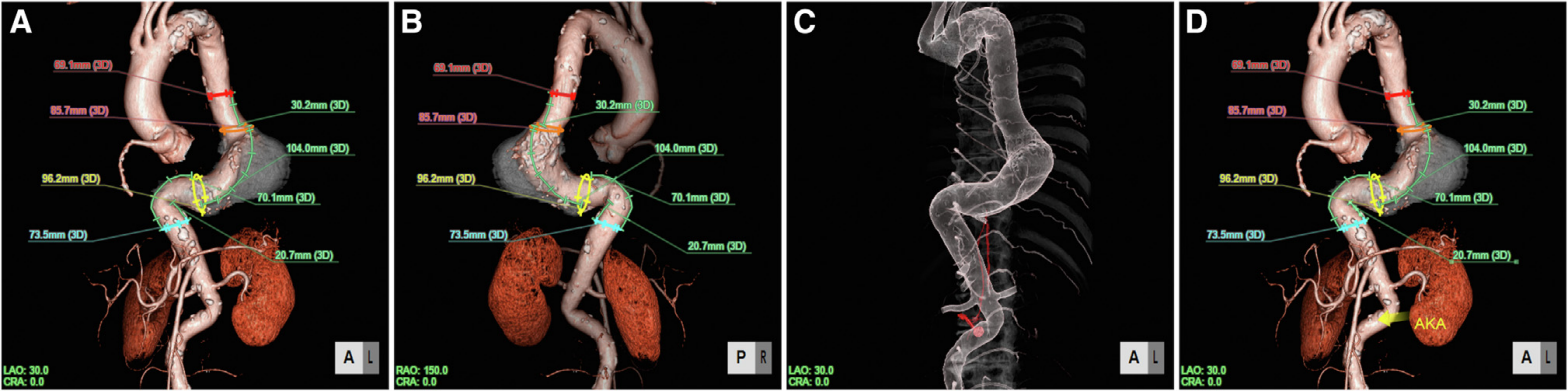
Volume-rendered images of computed tomography angiographies (CTAs) in a 70-year-old woman with aortic aneurysm. The volume-rendered images of standard CTA with thoracic endovascular aortic repair (TEVAR) planning are shown (A, B). The aneurysmal section is marked in *orange to yellow*, the proximal landing site of the TEVAR plan is *red*, and the distal site is *light blue*. The measured values represent the inner perimeter of the true lumen at each site. In the standard CTA, the Adamkiewicz artery (AKA) was not identified, and the distal landing was at zone 5, posing a potential risk of spinal cord ischemia (SCI) due to possible AKA coverage. (C) The AKA-specific CTA (AKA-CTA) is shown, revealing the AKA originating from the right L2 segmental artery (*red-colored vessel*). The TEVAR planners were informed of this L2 branching (indicated by a *yellow arrow* in panels A and B). Although the TEVAR plan remained unchanged, TEVAR planners reassessed the SCI risk as low.

**Figure E1:**
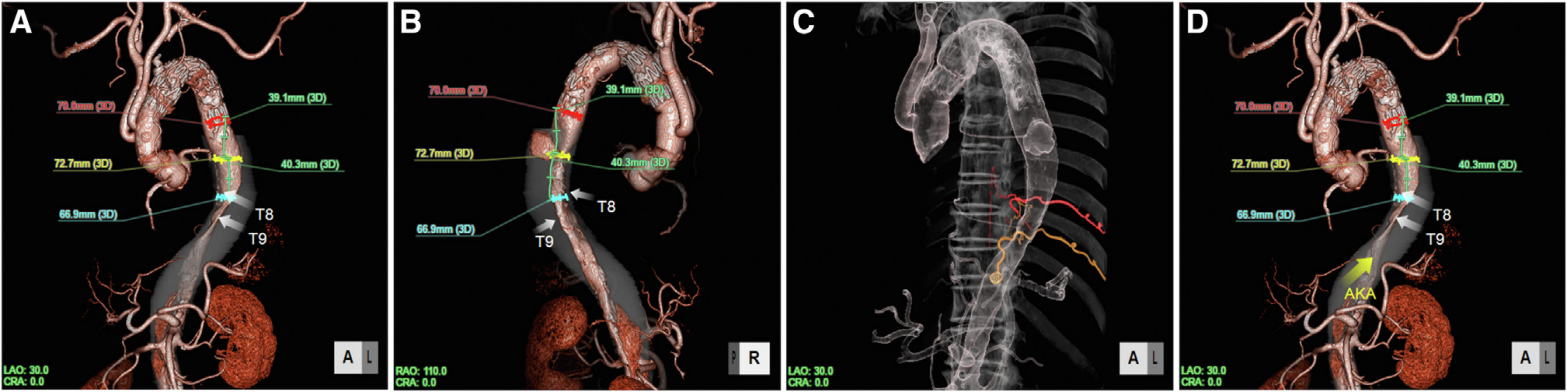
Volume-rendered images of computed tomography angiographies (CTAs) in a 70-year-old woman with recurrence aortic dissection. The volume-rendered images of standard CTA with thoracic endovascular aortic repair (TEVAR) planning are shown (A, B). The covered entry section is marked in *yellow*, the proximal landing site of the TEVAR plan is marked in *red*, and the distal site is marked in *light blue*. The measured values represent the inner perimeter of the true lumen at each site. In the standard CTA, the Adamkiewicz artery (AKA) was not identified, and the distal landing was at zone 5, posing a potential risk of spinal cord ischemia (SCI) due to possible AKA coverage. (C) The AKA-specific CTA (AKA-CTA) is shown, revealing the AKA originating from the left T9 segmental artery (*red-colored vessel*) with occlusion at the aortic branch and collateral pathways from the T10 segmental artery (*orange-colored vessel*). The TEVAR planners were informed of this T10 branching (indicated by a *yellow arrow* in panels A and B). Although the TEVAR plan remained unchanged, TEVAR planners reassessed the SCI risk as low.

**Figure E2:**
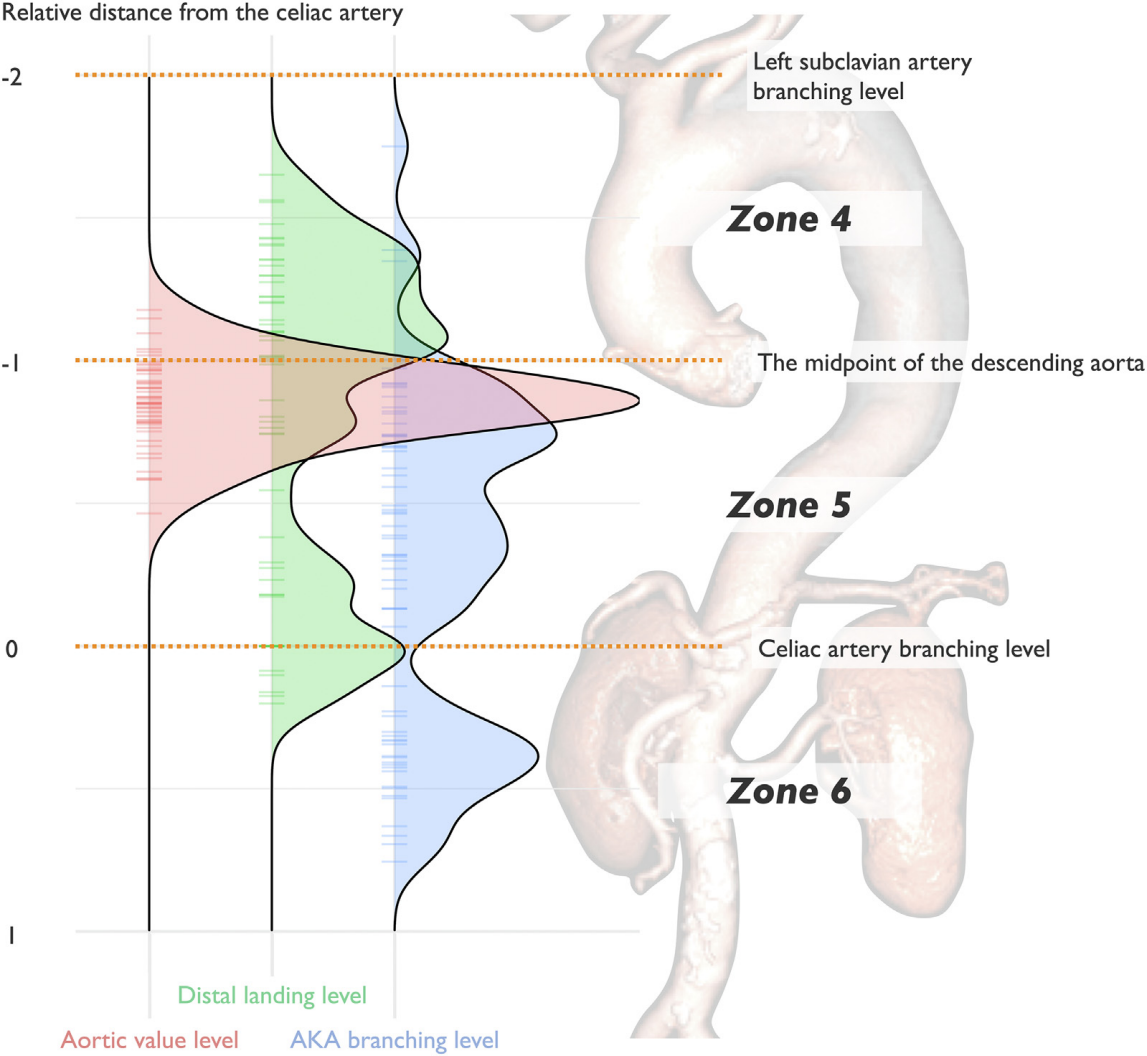
This ridgeline plot illustrates the positions of the Adamkiewicz artery (AKA) branching point, the distal landing zone of the thoracic endovascular aortic repair (TEVAR) plan, and the aortic valve level relative to the celiac artery bifurcation. The distances are expressed as relative distances, calculated by dividing the aortic distance from the celiac artery bifurcation to each site by the total distance from the celiac artery bifurcation to the midpoint of the descending aorta. In this scale, a relative distance of −1 corresponds to the midpoint of the descending aorta, whereas 0 indicates the celiac artery bifurcation. These positions align with the boundaries between zones 4 and 5 and between zones 5 and 6. The plot uses different colors to represent each anatomical location: the AKA branching level is shown in *blue*, the distal landing level in *green*, and the aortic valve level in *red*. The AKA and distal landing positions exhibit bimodal density curves, with fewer AKA branches near the celiac artery bifurcation. Additionally, the cross-sectional plane at the same horizontal level as the aortic valve roughly coincides with the boundary between zones 4 and 5 on the computed tomography scan. This suggests that if the TEVAR distal landing zone is designated at or beyond the aortic valve level, it will likely fall within zone ≥5, which may warrant AKA-specific computed tomography angiography. This could simplify evaluation without the need for precise identification of the descending aorta.

TEVAR planning was conducted for 57 patients (median age, 71 [62–80] years; 36 male patients) of 80 with discordant results between CTA and AKA-CTA; 23 cases were excluded as unsuitable for TEVAR on the basis of the morphologic characteristics of the aorta, of which 15, 2, and 6 were considered for open surgery, alternative endovascular interventions, and only observation, respectively. Proximal LZs ≥3 and distal LZs ≥5 were found in 72% (41/57) and 51% (29/57) of the cases, and the median cover length was 150 (100-150) mm. An SCI risk score of ≥1 was observed in 67% (38/57) cases. On the basis of additional AKA-CTA information, we adjusted the TEVAR planning or SCI risk score in 32% (18/57) of patients. In 2 patients, the grafts were shortened by 10 and 20 mm to prevent covering the AKA. The median SCI scores decreased in the remaining patients from 2 (1-3) to 1 (0-2) after AKA-CTA confirmed the uncovered AKA. Significantly more patients with informative AKA-CTA data had proximal LZ ≥ 3 (*P* = .02), distal LZ ≥ 5 (*P* < .001), and greater SCI scores (*P* < .001) (Table 3). Multivariate analysis selected the distal LZ ≥ 5 (odds ratio, 16; 95% confidence interval, 3.8-111; *P* < .001) as a significant independent factor for these changes. Figures 4and E1 show patients with informative AKA-CTA. The AKA was preserved in patients with a distal LZ ≤ 4; however, those with a distal LZ ≥ 5 were significantly more likely to cover the AKA (*P* < .001; Table E4). Table E5 and Figure E2 show distances along the aortic centerline from the celiac artery bifurcation and the relative distance from the middle aorta to the celiac artery bifurcation.

**Table 3:** Comparison of results influenced by changing TEVAR plan or SCI risk assessment after adding AKA-CTA data versus unchanged plan or risk assessment (noninformative AKA-CTA)

Variables	Overall n = 57	Informative n = 18	Noninformative n = 39	*P* value
Age, y	71 (62, 80)	71 (67, 80)	72 (60, 78)	.62
Male patient	36 (63)	10 (57)	26 (67)	.61
Body mass index, kg/m^2^	22.8 (21.0, 26.3)	22.0 (20.9, 24.0)	23.2 (21.3, 27.7)	.24
Disease				.39
AD	32 (56)	10 (56)	22 (56)	
TAA	22 (39)	6 (33)	16 (41)	
Other	3 (5)	2 (11)	1 (3)	
AKA findings				
Branching level				.19
T8	5 (9)	0 (0)	5 (13)	
T9	13 (23)	3 (17)	10 (26)	
T10	6 (11)	2 (11)	4 (10)	
T11	9 (16)	3 (17)	6 (15)	
T12	3 (5)	0 (0)	3 (8)	
L1	12 (21)	5 (28)	7 (18)	
L2	6 (11)	2 (11)	4 (10)	
Other	3 (5)	3 (17)	0 (0)	
Branching laterality, left	40 (70)	15 (83)	25 (64)	.25
Branching level, lumber	18 (32)	7 (39)	11 (28)	.62
Branching lumen, true	50 (88)	16 (89)	34 (87)	.33
Collateral pathway detection	12 (21)	5 (28)	7 (18)	.62
Other ARMA detection	11 (19)	5 (28)	6 (15)	.46
TEVAR planning				
Covered length, mm	150 (100, 150)	150 (150, 199)	150 (100, 150)	.11
Proximal landing zone ≥3	41 (72)	17 (94)	24 (62)	.02
Distal landing zone ≥5∗	29 (51)	16 (89)	13 (33)	<.001
Shaggy aorta	7 (12)	3 (17)	4 (10)	.80
Obstructed collateral source	7 (12)	2 (11)	5 (13)	1.0
SCI risk score	1 (0, 2)	2 (1, 3)	1 (0, 1)	<.001

Data are shown as medians with interquartile ranges and numbers of patients n (%). *TEVAR*, Thoracic endovascular aortic repair; *AKA-CTA*, Adamkiewicz artery−specific computed tomography angiography; *AD*, aortic dissection; *TAA*, thoracic aortic aneurysm; *ARMA*, anterior radiculomedullary artery; *SCI*, spinal cord ischemia.

∗ Multivariate analysis selected a distal landing zone of ≥5 as the sole independent factor that could predict informative AKA-CTA.

## Discussion

Among the 160 patients, standard CTA and AKA-CTA identified AKA in 95 (59.4%) and 155 (96.9%) patients, respectively. Standard CTA demonstrated a 50.0% (80/160) concordance with AKA-CTA.TEVAR plans or SCI risk scores were changed on the basis of AKA-CTA data for 32% (18/57) of patients with discordant AKA identification between CTAs. Multivariate analysis indicated that a distal LZ ≥ 5 was an independent predictor of these changes.

Although standard CTA identified the AKA in one half of the patients, which is consistent with a previous report,^16^ the present study differs. We included data from 3 CT scanners, incorporated the reference AKA-CTA findings, and targeted patients with aortic disease who might require intervention. In addition, AKA-CTA visualized the AKA in 97% of patients, which concurred with previous reports.^14^^,^^15^ AKA-CTA achieves high visualization performance using a low tube voltage,^14^ a sharp kernel with high spatial resolution,^15^ and bone-removal techniques,^20^ thus enhancing the contrast and vessel continuity within the intervertebral foramen. Therefore, standard CTA can be used to evaluate the AKA to some extent; however, AKA-CTA should be incorporated when the results are unclear or when a more precise assessment is needed.

Predicting SCI during TEVAR involves established risk factors.^7^^,^^8^^,^^21^ However, the role of AKA coverage alone in SCI prevention remains controversial.^7^ The primary scientific concept^22^ underpinning spinal cord blood flow in aortic treatment is the "collateral network," which suggests that focusing solely on a single AKA is less crucial than considering the overall balance of spinal cord blood flow. However, this concept does not imply that AKA evaluation is irrelevant. We showed that incorporating AKA-CTA into TEVAR planning for patients with a distal LZ ≥ 5 can significantly influence SCI risk assessment and treatment strategies. Therefore, determining the AKA branching level with selective use of AKA-CTA is crucial to accurately assess SCI risk and facilitate informed decision-making by surgeons and patients.^5^

We could generalize the following on the basis of our results. First, surgeons and radiologists can assess the AKA using standard CTA when planning aortic repair. A high PPV (82%) indicates that standard CTA is reliable when the AKA is visible, comparable with AKA-CTA. However, because of the low NPV (3%) of standard CTA, adding AKA-CTA is advisable to identify patients who might be at risk when the AKA is undetectable. For open aortic repair, precise identification of the AKA via AKA-CTA can facilitate effective reconstruction, minimizing the risk of SCI.^1^ In TEVAR cases with a distal LZ in zone 4, the AKA was preserved according to our results, reducing the necessity for additional AKA-CTA. Conversely, adding AKA-CTA becomes crucial when planning TEVAR with a distal LZ ≥ 5, aligning with a previous report^6^ highlighting the value of preoperative AKA evaluation.

This study has some limitations. Its retrospective, single-center design might need more generalizability. Our institution's strict focus on SCI prevention required us to minimize the area covered during TEVAR, which might not reflect broader clinical practices. Differences in AKA interpretation skills with CTAs across centers might limit the generalizability. Using a single device for TEVAR planning restricts applicability, as clinical practice typically involves various devices for personalized treatment (Appendix E2 provides information on detailed clinical treatments in the TEVAR planning group). However, this restriction ensures consistency within our study. High-urgency cases in which AKA-CTA was bypassed for immediate treatment were not included, potentially affecting the relevance of our findings in emergent TEVAR. This study does not rely on the actual clinical incidence of SCI (Appendix E2) as an outcome; instead, it is determined on the basis of simulations. Therefore, multicenter prospective studies are warranted.

In conclusion, although standard CTA identified AKA that was consistent with AKA-CTA findings in 50% of the cases, AKA-CTA provided value-added insights over standard CTA, particularly for thoracic endovascular aortic repair cases with ≥5 distal LZs, influencing treatment decisions or SCI risk assessments. However, randomized controlled studies using reference SCI outcomes are required to evaluate the real-world effects of selectively adding AKA-CTA over standard CTA to prevent SCI.

## Conflict of Interest Statement

All authors reported no conflicts of interest.

The *Journal* policy requires editors and reviewers to disclose conflicts of interest and to decline handling or reviewing manuscripts for which they may have a conflict of interest. The editors and reviewers of this article have no conflicts of interest.

## Figures and Tables

**Table E1:** Standard CT angiography scan parameters

Parameters	CT1	CT2	CT3
Image acquisition			
Scanner	Aquilion ONE (Canon)	Revolution CT (GE)	SOMATOM Force (Siemens)
Collimation, mm	80 × 0.5	128 × 0.625	2 × 192 × 0.6
Scan mode	Variable helical pitch scan	High-pitch helical scan	High-pitch scan/Single helical scan
Tube potential, kV	100, 120	100, 120	90, 100, 120
Rotation time, s	0.275	0.28	0.25
Pitch	1.188 (thorax), 0.813 (abdomen)	1.531	0.7-2.4
Auto exposure control setting	Volume EC, SD, 8.0 in, 5 mm	Smart mA, Noise index, 8.0	CARE Dose4D, Quality reference mAs, 180 mAs in 120 kV
Image reconstruction			
Slice thickness, mm	1	1.25	1
Slice interval, mm	1	1.25	1
Field of view, mm	400	400	400
Kernel with IR/DLIR strength	AiCE Body sharp Standard	Stnd, TrueFidelity high	Bv40, ADMIRE 3

*CT*, Computed tomography; *IR*, iterative reconstruction; *DLIR*, deep-learning image reconstruction.

**Table E2:** AKA-specific CT angiography scan parameters

Parameters	AKA-specific CT angiography
Image acquisition	
Scanner	SOMATOM Force (Siemens)
Collimation, mm	2 × 128 × 0.6
Scan mode	Dual-power helical scan
Tube potential, kV	70-100
Rotation time, s	0.5
Pitch	0.6
Auto exposure control setting	CARE Dose 4D, Quality reference mAs, 800 in 80 kV (noncontrast scan, 1600 in 80 kV (early- and late-arterial scan)
Image reconstruction	
Slice thickness, mm	0.75
Slice interval, mm	0.5
Field of view, mm	160
Kernel with IR strength	Bv59, ADMIRE 4

AKA, Adamkiewicz artery; CT, computed tomography; IR, iterative reconstruction.

**Table E3:** AKA distribution in AKA-specific CT angiography

Branching levels	Total n = 155	Right n = 39	Left n = 116
T5	1 (0.6)	0 (0)	1 (0.9)
T6	2 (1.3)	0 (0)	2 (1.7)
T7	5 (3.2)	2 (1)	3 (2.6)
T8	13 (8.4)	4 (3)	9 (7.8)
T9	34 (21.9)	11 (7)	23 (19.8)
T10	24 (15.5)	8 (5)	16 (13.8)
T11	26 (16.8)	5 (3)	21 (18.1)
T12	10 (6.5)	3 (2)	7 (6.0)
L1	27 (17.4)	4 (3)	23 (19.8)
L2	9 (5.8)	2 (1)	7 (6.0)
L3	2 (1.3)	0 (0)	2 (1.7)
Other	2 (1.3)	0 (0)	2 (1.7)

Data are presented as the number of patients with percentages in parentheses. *AKA*, Adamkiewicz artery; *CT*, computed tomography.

**Table E4:** Difference between the groups with and without AKA coverage in TEVAR planning

Variables	AKA uncovered n = 44	AKA covered n = 13	*P* value
Age, y∗	71 (62, 80)	72 (63, 79)	.78
Male	29 (66)	7 (54)	.64
Body mass index, kg/m^2^∗	23.1 (21.0, 26.7)	22.6 (21.0, 24.5)	.41
AKA findings			
Branching level			.56
T8	3 (7)	2 (15)	
T9	8 (18)	5 (39)	
T10	4 (9)	2 (15)	
T11	8 (18)	1 (8)	
T12	2 (5)	1 (8)	
L1	10 (23)	2 (15)	
L2	6 (14)	0 (0)	
Other	3 (7)	0 (0)	
Branching laterality, left	33 (75)	7 (54)	.26
Branching level, lumbar	16 (36)	2 (15)	.28
Branching lumen, true	37 (84)	13 (100)	.25
Collateral pathway detection	8 (18)	4 (31)	.56
Other ARMA detection	7 (16)	4 (31)	.43
TEVAR planning			
Covered length, mm∗	150 (150, 150)	100 (100, 163)	.31
Proximal landing zone ≥3	29 (66)	12 (92)	.13
Distal landing zone ≥5	15 (34)	13 (100)	<.001
Distal landing zone			<.001
4	28 (64)	0 (0)	
5	14 (32)	10 (77)	
6	2 (5)	3 (23)	
Distance from the celiac artery			
Distal landing, mm∗	−148.8 (−169.2, −109.6)	0 (−22.0, 0)	<.001
AKA branching level, mm∗	−35.8 (−81.6, 44.1)	−70.5 (−91.0, −21.0)	.10

Except where indicated, data represent the numbers of patients, with percentages in parentheses. *AKA*, Adamkiewicz artery; *TEVAR*, thoracic endovascular aortic repair; *ARMA*, anterior radiculomedulary artery.

∗ Data are presented as medians with interquartile in parentheses.

**Table E5:** Comparison of distances from the celiac artery bifurcation between informative and noninformative AKA-specific CT angiography groups

Variables	Overall n = 57	Informative n = 18	Noninformative n = 39	*P* value
Distance from the celiac artery, mm				
Midpoint of the descending aorta	−130.8 (−142.8, −123.5)	−142.9 (−151.3, −126.4)	−127.0 (−137.1, −123.1)	.02
Aortic valve cross-sectional level in the descending aorta	−112.5 (−126.0, −96.5)	−116.8 (−131.4, −102.6)	−110.0 (−124.5, −96.2)	.39
Distal landing level	−133.0 (−156.0, −25.0)	−86.2 (−127.5, −6.2)	−143.0 (−163.0, −32.0)	.08
AKA branching level	−44.5 (−89.2, 42.0)	−39.0 (−68.5, 44.5)	−59.5 (−91.0, 39.2)	.48
Relative distance∗				
Distal landing level	−1.0 (−1.2, −0.2)	−0.6 (−0.8, 0.0)	−1.1 (−1.3, −0.3)	.03
AKA branching level	−0.3 (−0.7, 0.3)	−0.3 (−0.5, 0.4)	−0.5 (−0.7, 0.3)	.31

Data are presented as medians with interquartile ranges in parentheses. *AKA*, Adamkiewicz artery.

∗ Relative distance was calculated as the position along the aorta from the celiac artery bifurcation, normalized by the total length to the midpoint of the descending aorta. A value of 0 indicates the site is directly above the celiac artery, whereas −1 corresponds to the midpoint of the descending aorta.

## Appendix E1 Details of Computed Tomography Angiography (CTA) Acquisition and Reconstruction

We performed standard-body CTA using 3 computed tomography scanners: 320-row (Aquilion ONE; Canon Medical Systems), 256-row (Revolution CT; GE HealthCare), and 196-row dual-source scanners (SOMATOM Force; Siemens). Either 320 or 370 mg I/mL contrast (OptiRay 320 [Guerbet Japan] or Iopamiron 370 [Bayer Yakuhin]) was injected at a body weight−adjusted rate of 18.5 mg I/kg/s over 20 to 25 seconds, followed by a saline flush from a dual-head power injector (Dual Shot GX7; Nemoto-Kyorindo). The timing of image acquisition was determined using bolus tracking. Images were reconstructed using deep learning−based methods (AiCE, Canon or TrueFidelity; GE HealthCare) or iterative reconstruction methods (ADMIRE strength 4; Siemens). The slice thickness was 1 to 1.25 mm, and the field of view was 400 cm. The detailed scan and reconstruction parameters are listed in Table E1.

We used a 196-row dual-source CT scanner (SOMATOM Force) in the dual-power mode with a low-kV tube potential for Adamkiewicz artery (AKA)-CTA. The optimal tube potential and current were selected using an automatic selection algorithm (CARE kV combined with CARE dose 4D; Siemens), according to the individual topogram. A noncontrast scan initially proceeded, followed by early and late arterial phase scans after contrast injection separated by 7 seconds. The noncontrast scan created a bone mask for bone-subtracted CTAs, whereas the late arterial phase facilitated distinguishing radiculomedullary arteries from veins. The scan extended from the aortic arch to the fourth lumbar vertebra (Table E2). Contrast administration was performed using a dual-head power injector with either 350 or 370 mg I/mL contrast material (OptiRay or Iopamiron) at a body weight−adjusted rate of 26 mg I/kg/s, followed by a saline flush. The scan timing was optimized for peak contrast at the T10 level of the descending aorta as determined using the timing-bolus method. AKA-CTA images were reconstructed with a 160-cm field of view and 0.75/0.5 mm thickness/interval using a sharp filter kernel (Bv59) and iterative reconstruction (ADMIRE strength 4). We generated a bone subtraction image using a 3D workstation (Ziostation2; Ziosoft).

## Appendix E2 Treatment Summary of the Thoracic Endovascular Aortic Repair (TEVAR) Planning Group on the Basis of Actual Clinical Records

The vascular team carefully reviewed the selection and planning of TEVAR devices in clinical practice, integrating data from both the standard computed tomography angiography (CTA) and the Adamkiewicz artery−specific CTA. Patients were informed of the team's conclusions and the potential risks associated with the proposed treatment plan. On the basis of this comprehensive assessment, a final decision was made regarding the most appropriate course of action. Of the 57 cases evaluated, 32 were selected for TEVAR, 6 for open repair, and 19 for observation. The stent graft devices used included 12 GORE TAG conformable thoracic stent grafts, 7 Terumo Relay series, 7 Cook Zenith series, 4 Medtronic Valiant Captiva, 1 Kawasumi Najuta, and 1 Medtronic Valiant Navion. Motor-evoked potential was monitored in all cases. Cerebrospinal fluid drainage was not performed in any patient, except in one case. Despite a reduction in intraoperative motor-evoked potential monitoring in three patients, none of the patients in the study group exhibited posttreatment symptoms of spinal cord ischemia.
